# School environmental contamination of methicillin-sensitive *Staphylococcus aureus* as an independent risk factor for nasal colonization in schoolchildren: An observational, cross-sectional study

**DOI:** 10.1371/journal.pone.0208183

**Published:** 2018-11-30

**Authors:** Jialing Lin, Ting Zhang, Chan Bai, Jianping Liang, Jiaping Ye, Zhenjiang Yao

**Affiliations:** 1 Department of Epidemiology and Health Statistics, School of Public Health Guangdong Pharmaceutical University, Guangzhou, China; 2 Department of Health Education, Guangzhou Primary and Secondary School Health Promotion Center, Guangzhou, China; Boston University School of Public Health, UNITED STATES

## Abstract

**Objective:**

We aim to assess the similarities of proportional, phenotypic, and molecular characteristics between the school environment and schoolchildren on methicillin-sensitive *S*. *aureus* (MSSA) isolates.

**Methods:**

A cross-sectional study was conducted between March 2016 and August 2016 in eight elementary schools in Guangzhou, China. Nasal swabs from students and environmental swabs from school environments were collected. Univariate and multivariate logistic regression analyses under a multistage stratified cluster cross-sectional survey design were performed to access the prevalence relationship and influencing factors, respectively. Phenotypic and molecular characterizations of MSSA isolates were conducted using the Kirby-Bauer disk diffusion method and polymerase chain reaction assays, respectively.

**Results:**

In total, 1705 schoolchildren and 1240 environmental samples from 40 classes in eight elementary schools obtained between March and August 2016 were include in this study. The rates of MSSA prevalence among schoolchildren and the environment were 11.44% (195/1705) and 4.60% (57/1240), respectively. The odds ratios and 95% confidence intervals (CIs) on the prevalence of MSSA isolates were 1.11 (95% CI, 1.05–1.29; *P* = 0.010) and 1.04 (95% CI, 1.01–1.07; *P* = 0.003) for the school or class environment and students, respectively. Similar phenotypic and molecular characteristics were identified between schoolchildren and the environment. A cause and effect relationship could not be established because the study design was cross-sectional.

**Conclusions:**

Because of the cross-sectional design, we can reveal the association between school environment and schoolchildren on MSSA, but it is not a cause and effect relationship.

## Introduction

*Staphylococcus aureus* (*S*. *aureus*) has become one of the leading causes of bacteremia with a 20–30% mortality rate [1]. Greater than 80% of *S*. *aureus* isolates can cause skin and soft tissue infection in healthcare settings, and these infections are identical to the colonization strain, indicating that *S*. *aureus* colonization played a significant role in subsequent infections [2]. *S*. *aureus* commonly colonizes the skin and mucosal surfaces of humans, and the anterior nares are reported to be its most common ecological niche [3]. Currently, the prevalence of *S*. *aureus* colonization and infection in community settings has increased [4]. A study [5] demonstrated high proportions of *S*. *aureus* nasal colonization in schoolchildren.

Additionally, the environment plays an important role in *S*. *aureus* transmissions and infections not only in healthcare settings [6] but also in some high-risk community settings, including households [7, 8], prisons [9, 10], and public transportation [11, 12]. In an Iranian intensive care unit, *S*. *aureus* strains isolated from the air, environment and patients who were colonized or infected with *S*. *aureus* often exhibited the same molecular characteristics [13]. The study also revealed that isolates from patients and the environment were typically indistinguishable. With regard to community settings, environmental contamination has been increasingly recognized for its possible role in *S*. *aureus* transmission within schoolchildren [14]. An American study reported homology of *S*. *aureus* strains between dental students and environmental surfaces in the dental school clinic [15]. A Mexican study also identified homologous *S*. *aureus* strains between students and their mobile phones [16].

Schools with forced crowding or increased density of human contacts, could also possibly provide the opportunity for the transmission of *S*. *aureus* [17]. Schoolchildren are more likely to be colonized with *S*. *aureus* compared with other populations [18]. There is a paucity of data regarding *S*. *aureus* nasal colonization in both schoolchildren and school environment in previous studies, none of which specifically referred to methicillin-sensitive *S*. *aureus* (MSSA). Therefore, we conducted a multistage stratified cluster cross-sectional survey designed to determine the dose-response, phenotypic, and molecular relationship of MSSA isolates between schoolchildren and the school environment. Moreover, we also elucidated the influencing factors of MSSA nasal colonization in schoolchildren.

## Materials and methods

This observational study followed the Strengthening the Reporting of Observational Studies in Epidemiology (STROBE) Statement: guidelines for reporting observational studies (S1 Table), the analysis plan (S1 File), and the protocol (http://dx.doi.org/10.17504/protocols.io.vcce2sw). The dataset (S1 Data) and questionnaire (S2 File) of this study can be found in the supplementary files.

### Setting and study design

This multistage stratified cluster cross-sectional study was conducted from March to August 2016 in eight elementary schools in Guangzhou, China. Schoolchildren were randomly selected using a multistage stratified cluster sampling design. First, we randomly selected three administrative districts from the urban region (Haizhu, Huangpu, and Huadu District) and one administrative district (Conghua District) from the rural region. Second, two elementary schools from each administrative district and eight schools were included. The buildings of these schools were constructed from reinforced concrete and brick and surrounded by trees. Third, the number of classes in grades four and five were proportional and randomly selected. All schoolchildren who obtained consent from their legal representatives were included in the study. Schoolchildren from grades four and five were selected given their better compliance and lighter academic burden in China. Schoolchildren who were absent were excluded. The study was approved by the Ethics Committee of Guangdong Pharmaceutical University, and performed in accordance with the approved guidelines of the Declaration of Helsinki. All legal representatives of participants signed an informed written consent form.

We selected classrooms, toilets, and public places of each school for environmental sampling. We sampled five locations (floor, light switch, door handle, desk, chair, and air) of the classroom, four locations (floor, light switch, door handle, faucet, and toilet flush handle) of the two toilets (men’s and women’s), and three locations (handle of stairs, floor of stairs, and floor of corridor) of the public place from each class. Overall, we obtained 31 samples from each class. These locations are frequently touched by people, easily contacted by skin, and amenable to cleaning and disinfection. To avoid bias of sampling time, we performed sampling between two and four in the afternoon on weekdays.

### Questionnaires and sampling

We collected information through questionnaires answered by parents of schoolchildren. Information regarding demographic characteristics, personal characteristics, characteristics of family members, and household characteristics was collected. The questionnaire was first prepared in English and then translated into the local language (Chinese), and back to English to ensure consistency. Sterile swabs (BBL Culture Swab; Becton Dickinson) moistened with sterile saline water were used to sample both nasal vestibules of the schoolchildren and surfaces of environmental locations by trained personnel. Floor, desks, and chairs were swiped in an approximately 10×10 cm^2^ sample area given their regular surfaces. The entire surfaces of light switches, handles, and toilet faucets were swiped given their irregular surfaces. Each swab was placed into a sterile tube with 7.5% sodium chloride broth, and the tubes were transported to the laboratory immediately.

We used the natural sedimentation method to collect air samplings. We placed five mannitol salt agar plates on five desktops in each classroom (one plate in the center and four at the four corners of the classroom) ten minutes after the classroom became empty. After ten minutes of exposure, we closed the cover of plates and transferred them to the laboratory for further analyses. To ensure the acquisition of quality data and the quality of sampling, all investigators were trained by the principal investigator for three days before the survey, and random assessments were performed by the principal investigator. We conducted sampling during the afternoon (2–4 p.m.) on weekdays to avoid the bias of the time.

### Laboratory methods

Details on our laboratory methods can be found in our previous study [19]. Air samples were incubated for 48 hours at 37±1°C. The swabs were transferred to mannitol salt agar plates for a 24-hourincubation after a first 24-hour incubation at 37±1°C. Samples, including air samples, were identified as *S*. *aureus* isolates through specific colony morphology and positivity for Gram-staining, catalase reaction, hemolysis test, DNase test, and coagulase test.

All *S*. *aureus* isolates underwent cefoxitin susceptibility testing using the Kirby-Bauer disk diffusion method, following the Clinical and Laboratory Standards Institute guidelines from 2015. *S*. *aureus* isolates that were sensitive to cefoxitin were identified as MSSA isolates. All MSSA isolates were then subject to specific antibiotic susceptibility testing using the Kirby-Bauer disk diffusion method and 11 antimicrobial agents: cefoxitin, penicillin, linezolid, gentamicin, teicoplanin, erythromycin, trimethoprim-sulfamethoxazole, moxifloxacin, rifampin, chloramphenicol, tetracycline, and clindamycin.

DNA was obtained from all MSSA isolates using the sodium dodecyl sulfate method [20] and were further tested to confirm the presence toxin genes [*Tst*, *Eta*, *Etb*, and Panton-valentine leukocidin (*Pvl*)] [21, 22] using polymerase chain reaction (PCR) assays.

Multilocus sequence typing (MLST) was performed using previously published primers and conditions [23]. Allelic profiles, clonal complex (CC) types, and sequence types (STs) were assigned using the MLST database (http://www.mlst.net). Singletons or members of a clonal complex were determined using a related sequence types algorithm (http://eburst.mlst.net). Dendrogram analysis was performed based on STs to determine the clonal relatedness and potential epidemiologic origin.

### Definitions of obesity

Anthropometric measurements were obtained using standardized techniques and calibrated equipment. Schoolchildren were weighted to the nearest 0.1 kg in light indoor clothing and bare feet or with stockings. Height was measured using a stadiometer. Schoolchildren stood in erect posture without shoes, and the results were recorded to the nearest 0.1 cm. Measures were obtained twice, and the average was considered in the analysis. Body mass index (BMI) was calculated as the ratio of weight in kilograms to the square of height in meters (kg/m^2^). Age- and gender-specific BMI cutoff points recommended by the Working Group on Obesity in China were used to define obesity [24].

### Statistical analysis

The significance of the difference between proportions was assessed using Pearson’s Chi-square test for categorical variables. There were two clusters in the study; a class cluster and a school cluster. We drew linear prediction plots to intuitively elucidate the dose-response relationship of MSSA isolates between nasal colonization among schoolchildren and environment in class or school clusters. The association between influencing factors and MSSA nasal colonization was examined using multivariable logistic regression models. We performed multivariable logistic regression analysis of all variables with a *P*-value of <0.2 indication significance. We removed variables that were not significant at this level. All statistical measures were estimated using survey data analysis methods (SVY commands) from STATA package. A two-sided *P*-value for statistical significance was defined as *P*-value of < 0.05. All analyses were performed using STATA 14.2 (College Station, Texas, USA).

## Results

### Prevalence of MSSA in both schoolchildren and the environment

In total, 1705 schoolchildren and 1240 environmental samples from 40 classes in eight elementary schools were included in the study. The prevalence of MSSA nasal colonization among schoolchildren was 11.44% (195/1705). A significant difference in MSSA (school: χ^2^ = 53.01, *P*<0.001; class: χ^2^ = 95.23, *P*<0.001) nasal colonization was noted among schoolchildren in different schools and classes. More details of the characteristics of participants are presented in Table 1.

**Table 1:** Influencing factors of MSSA nasal colonization among elementary students, Guangzhou, 2016.

Influencing factors	MSSA carriers (%)	Non-MSSA carriers (%)	Univariate logistic		Multivariate logistic	
			*P*	OR (95% CI)	*P*	OR (95% CI)
**Demographic characteristics**						
Gender (male)	121 (62.05)	795 (52.65)	0.123	0.66 (0.33–1.32)	0.184	0.69 (0.31–1.54)
Age, year (≤10)	118 (60.51)	915 (60.60)	0.944	0.99 (0.52–1.89)		
Region (urban)	174 (89.23)	1225 (81.13)	0.190	0.57 (0.17–1.96)	0.274	0.66 (0.20–2.20)
**Personal characteristics (within a year)**						
Obese	22 (13.17)	92 (6.91)	0.029	2.01 (1.72–5.59)	0.086	1.96 (0.79–4.87)
Antibiotic use	102 (52.58)	711 (47.24)	0.359	1.20 (0.62–2.30)		
Skin infection	93 (47.69)	740 (49.20)	0.975	0.99 (0.50–1.98)		
Allergic diseases	59 (30.26)	296 (19.69)	0.188	1.80 (0.50–6.55)	0.246	1.75 (0.40–7.74)
Upper respiratory tract infection	121 (62.37)	928 (61.70)	0.881	1.04 (0.36–3.00)		
Surgical intervention	12 (6.15)	76 (5.04)	0.850	1.08 (0.22–5.37)		
Outpatient service	121 (62.05)	817 (54.29)	0.194	1.30 (0.72–2.37)	0.504	1.15 (0.55–2.41)
Hospitalization	6 (3.08)	55 (3.64)	0.873	0.87 (0.03–27.26)		
Shared towel with others	47 (24.10)	387 (25.77)	0.746	1.04 (0.67–1.61)		
Had physical exercise every week	186 (96.88)	1420 (96.08)	0.155	1.96 (0.54–7.14)	0.171	1.84 (0.53–6.41)
Frequency of hand washing everyday (>6)	62 (32.12)	442 (30.25)	0.689	1.11 (0.42–2.90)		
Hand washing after using toilets (always)	110 (56.99)	824 (56.09)	0.896	0.98 (0.54–1.79)		
Way of going to school (public transportation)	16 (8.60)	168 (11.58)	0.576	0.77 (0.14–4.21)		
**Characteristics of family members (within a year)**						
Antibiotic use	64 (32.99)	471 (31.36)	0.214	0.86 (0.60–1.24)		
Skin infection	73 (37.63)	552 (36.82)	0.864	0.98 (0.65–1.48)		
Allergic disease	27 (13.92)	257 (17.13)	0.270	0.82 (0.46–1.46)		
Upper respiratory tract infection	99 (51.03)	774 (51.50)	0.709	0.92 (0.42–2.02)		
Hospitalization	35 (18.04)	251 (16.72)	0.782	1.08 (0.37–3.19)		
Healthcare workers	11 (5.67)	164 (10.93)	0.028	0.45 (0.26–0.81)	0.055	0.43 (0.18–1.05)
Smoking	100 (51.28)	839 (55.71)	0.366	0.86 (0.49–1.51)		
**Household characteristics**						
Family size (>3)	138 (71.88)	1105 (74.26)	0.783	0.91 (0.25–3.30)		
Single child	80 (41.67)	579 (39.89)	0.746	1.08 (0.46–2.54)		
Live with mammalian pets	48 (24.62)	357 (23.80)	0.937	1.02 (0.39–2.64)		

OR, odds ratio; CI, confidence interval.

The prevalence of MSSA environmental contamination was 4.60% (57/1240). Significant differences in MSSA (school: χ^2^ = 21.69, *P* = 0.003; class: χ^2^ = 89.57, *P*<0.001; location: χ^2^ = 33.06, *P* = 0.002) environmental contamination were noted in different schools, classes, and locations. However, no significant differences in MSSA (χ^2^ = 4.66, *P* = 0.097) environmental contamination were noted in different places. A more specific distribution of MSSA isolates in the environment is presented in Table 2.

**Table 2:** Distribution of MSSA isolates in the school environment, Guangzhou, 2016.

Location	Number of samples	Positive (%)	95% Confidence interval (%)
Classroom	760	35 (4.61)	3.32–6.35
Floor	40	5 (12.50)	5.09–27.56
Light switch	80	2 (2.50)	0.61–9.72
Door handle	40	2 (5.00)	1.18–18.87
Desk	200	16 (8.00)	4.94–12.71
Chair	200	7 (3.50)	1.67–7.20
Air	200	3 (1.50)	0.48–4.59
Toilet	320	10 (3.13)	1.68–5.72
Floor	40	5 (12.50)	5.09–27.56
Light switch	80	0 (0.00)	-
Door handle	40	0 (0.00)	-
Faucet	80	3 (3.75)	1.18–1.12
Toilet flush handle	80	2 (2.50)	0.60–9.71
Public place	160	12 (7.50)	4.28–10.81
Handle of stairs	80	7 (8.75)	4.16–17.48
Floor of stairs	40	2 (5.00)	1.18–18.88
Floor of corridor	40	3 (7.50)	2.32–21.71
Total	1240	57 (4.60)	3.56–5.92

### Influencing factors of MSSA nasal colonization among schoolchildren

We found that obese [odds ratio (OR), 2.01; 95% confidence interval (CI), 1.72–5.59; *P* = 0.029] and family members who were healthcare workers (OR, 0.45; 95% CI, 0.26–0.81; *P* = 0.028) were associated with MSSA nasal colonization among schoolchildren.

To account for the potential confounding factors among influencing factors, we further analyzed the relationship between the potential predictors using a multivariate logistic regression model. This model demonstrated that when controlling for the effects of the other influencing factors (gender, region, allergic diseases, outpatient service, and had physical exercise every week), the relationships found in the univariable analyses changed. Obese and family members who were healthcare workers were no longer associated with MSSA nasal colonization. More specific details are presented in Table 1.

### Relationship of MSSA isolates between schoolchildren and the environment

A positive relationship between MSSA isolates from the school or class environment and schoolchildren was observed. ORs and 95% CIs for the school or class environment and schoolchildren on the prevalence of MSSA isolates were 1.11 (95% CI, 1.05–1.29; *P* = 0.010) and 1.04 (95% CI, 1.01–1.07; *P* = 0.003), respectively.

**Fig 1:**
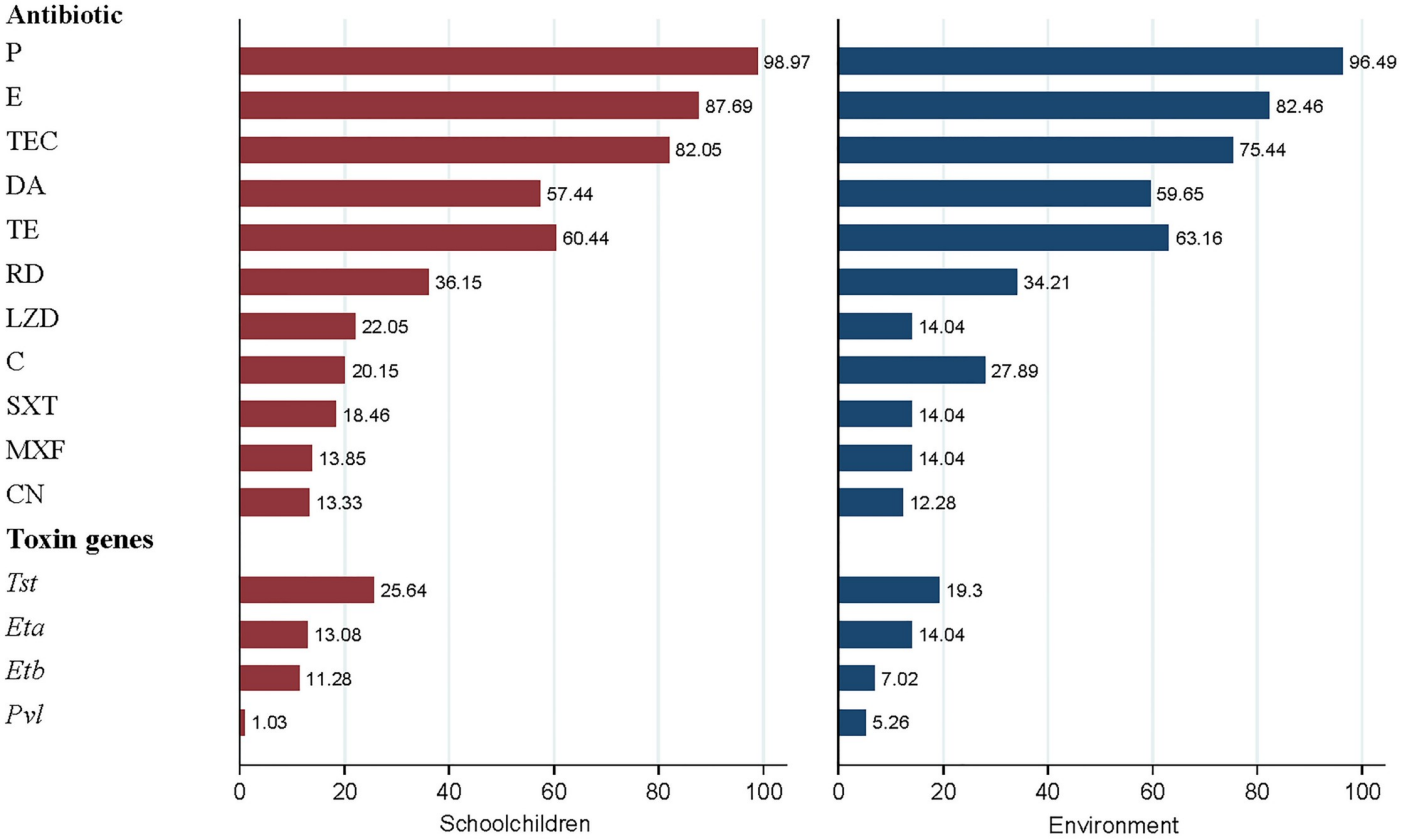
Prevalence of antibiotic resistance and genetic carriage in MSSA isolates between schoolchildren and environment, Guangzhou, 2016. P, Penicillin; E, Erythromycin; TEC, Teicoplanin; DA, Clindamycin; TE, Tetracycline; RD, Rifampin; LZD, Linezolid; C, Chloramphenicol; SXT, Trimethoprim-sulfamethoxazole; MXF, Moxifloxacin; CN, Gentamicin.

The proportions of antibiotic resistance among MSSA isolates in schoolchildren and the environment were not significantly different. In total, 84.62%, 67.70%, 54.37%, 45.14%, 24.11%, 8.73%, and 0.52% of MSSA isolates from schoolchildren were resistant to three, four, five, six, seven, eight, and nine antimicrobial agents, respectively. In total, 73.58%, 59.54%, 57.79%, 56.04%, 20.95%, 15.69%, 6.92%, and 3.41% of MSSA isolates from the environment were resistant to three, four, five, six, seven, eight, nine, and ten antimicrobial agents, respectively (Fig 1).

Among the proportions of all toxin genes, no gene was statistically significant between MSSA isolates from schoolchildren and the environment. More specific details are presented in Fig 1.

There were 22 CC types and 71 STs among all MSSA isolates in this study. The five most predominant CC types of MSSA isolates among schoolchildren were CC30 (13.33%, 26/195), CC6 (11.79%, 23/195), CC45 (11.79%, 23/195), CC188 (11.28%, 22/195), and CC121 (8.72%, 17/195). The five most predominant CC types of MSSA isolates in the environment were CC30 (19.30%, 11/57), CC188 (19.30%, 11/57), CC6 (14.04%, 8/57), CC5 (10.53%, 6/57), and CC398 (10.53%, 6/57). The proportions of different CC types of MSSA isolates between schoolchildren and the environment exhibited no significant differences. The five most predominant STs of MSSA isolates from schoolchildren were ST45 (10.26%, 20/195), ST6 (7.69%, 15/195), ST188 (7.69%, 15/195), ST30 (6.67%, 13/195), and ST7 (4.62%, 9/195). The five most predominant STs of MSSA isolates from the environment were ST30 (14.04%, 8/57), ST188 (14.04%, 8/57), ST6 (12.28%, 7/57), ST398 (8.77%, 5/57), ST5 (5.26%, 3/57), and ST217 (5.26%, 3/57). There was no significant ST of MSSA isolates between schoolchildren and the environment. Note that ST5, ST6, ST25, ST30, ST45, ST59, ST188, ST217, ST398, ST489, ST535, ST573, ST944, ST946, ST950, ST1295, and ST2114 were identified in MSSA isolates from both schoolchildren and the environment. More specific details are presented in Table 3.

**Table 3:** MLST distribution of MSSA isolates, Guangzhou, 2016 [n (%)].

CC type				MLST			
CC	Student	Environment	Total	ST	Student	Environment	Total
CC1	9 (4.62)	1 (1.75)	10 (3.97)	ST1	5 (2.56)	0 (0.00)	5 (1.98)
				ST573	2 (1.03)	1 (1.75)	3 (1.19)
				ST2518	1 (0.51)	0 (0.00)	1 (0.40)
				ST2592	1 (0.51)	0 (0.00)	1 (0.40)
CC5	16 (8.21)	6 (10.53)	22 (8.73)	ST5	5 (2.56)	3 (5.26)	8 (3.17)
				ST194	1 (0.51)	0 (0.00)	1 (0.40)
				ST627	2 (1.03)	0 (0.00)	2 (0.79)
				ST935	1 (0.51)	0 (0.00)	1 (0.40)
				ST950	2 (1.03)	1 (1.75)	3 (1.19)
				ST965	0 (0.00)	1 (1.75)	1 (0.40)
				ST1018	0 (0.00)	1 (1.75)	1 (0.40)
				ST1317	2 (1.03)	0 (0.00)	2 (0.79)
				ST1409	2 (1.03)	0 (0.00)	2 (0.79)
				ST1863	1 (0.51)	0 (0.00)	1 (0.40)
CC6	23 (11.79)	8 (14.04)	31 (12.30)	ST6	15 (7.69)	7 (12.28)	22 (8.73)
				ST932	2 (1.03)	0 (0.00)	2 (0.79)
				ST2114	6 (3.08)	1 (1.75)	7 (2.78)
CC7	9 (4.62)	0 (0.00)	9 (3.57)	ST7	9 (4.62)	0 (0.00)	9 (3.57)
CC8	3 (1.54)	0 (0.00)	3 (1.19)	ST630	2 (1.03)	0 (0.00)	2 (0.79)
				ST1821	1 (0.51)	0 (0.00)	1 (0.40)
CC10	4 (2.05)	0 (0.00)	4 (1.59)	ST10	4 (2.05)	0 (0.00)	4 (1.59)
CC15	6 (3.08)	0 (0.00)	6 (2.38)	ST15	5 (2.56)	0 (0.00)	5 (1.98)
				ST1906	1 (0.51)	0 (0.00)	1 (0.40)
CC22	4 (2.05)	3 (5.26)	7 (2.78)	ST217	4 (2.05)	3 (5.26)	7 (2.78)
CC25	3 (1.54)	1 (1.75)	4 (1.59)	ST25	3 (1.54)	1 (1.75)	4 (1.59)
CC30	26 (13.33)	11 (19.30)	37 (14.68)	ST30	13 (6.67)	8 (14.04)	21 (8.33)
				ST285	1 (0.51)	0 (0.00)	1 (0.40)
				ST434	1 (0.51)	0 (0.00)	1 (0.40)
				ST435	1 (0.51)	0 (0.00)	1 (0.40)
				ST452	1 (0.51)	0 (0.00)	1 (0.40)
				ST484	2 (1.03)	0 (0.00)	2 (0.79)
				ST535	2 (1.03)	2 (3.51)	4 (1.59)
				ST1386	1 (0.51)	0 (0.00)	1 (0.40)
				ST1478	3 (1.54)	0 (0.00)	3 (1.19)
				ST2102	0 (0.00)	1 (1.75)	1 (0.40)
				ST2121	1 (0.51)	0 (0.00)	1 (0.40)
CC45	23 (11.79)	3 (5.26)	26 (10.32)	ST45	20 (10.26)	1 (1.75)	21 (8.33)
				ST108	1 (0.51)	0 (0.00)	1 (0.40)
				ST396	0 (0.00)	1 (1.75)	1 (0.40)
				ST1017	1 (0.51)	0 (0.00)	1 (0.40)
				ST1891	1 (0.51)	1 (1.75)	2 (0.79)
CC50	1 (0.51)	0 (0.00)	1 (0.40)	ST50	1 (0.51)	0 (0.00)	1 (0.40)
CC59	9 (4.62)	1 (1.75)	10 (3.97)	ST59	6 (3.08)	1 (1.75)	7 (2.78)
				ST338	1 (0.51)	0 (0.00)	1 (0.40)
				ST537	1 (0.51)	0 (0.00)	1 (0.40)
				ST3069	1 (0.51)	0 (0.00)	1 (0.40)
CC72	3 (1.54)	0 (0.00)	3 (1.19)	ST72	3 (1.54)	0 (0.00)	3 (1.19)
CC88	1 (0.51)	0 (0.00)	1 (0.40)	ST2252	1 (0.51)	0 (0.00)	1 (0.40)
CC101	1 (0.51)	0 (0.00)	1 (0.40)	ST812	1 (0.51)	0 (0.00)	1 (0.40)
CC121	17 (8.72)	3 (5.26)	20 (7.94)	ST120	1 (0.51)	0 (0.00)	1 (0.40)
				ST121	6 (3.08)	0 (0.00)	6 (2.38)
				ST488	0 (0.00)	1 (1.75)	1 (0.40)
				ST489	1 (0.51)	1 (1.75)	2 (0.79)
				ST837	2 (1.03)	0 (0.00)	2 (0.79)
				ST946	6 (3.08)	1 (1.75)	7 (2.78)
				ST2798	1 (0.51)	0 (0.00)	1 (0.40)
CC182	3 (1.54)	2 (3.51)	5 (1.98)	ST944	3 (1.54)	1 (1.75)	4 (1.59)
				ST2694	0 (0.00)	1 (1.75)	1 (0.40)
CC188	22 (11.28)	11 (19.30)	33 (13.10)	ST188	15 (7.69)	8 (14.04)	23 (9.13)
				ST833	1 (0.51)	0 (0.00)	1 (0.40)
				ST1047	0 (0.00)	1 (1.75)	1 (0.40)
				ST1295	3 (1.54)	1 (1.75)	4 (1.59)
				ST1724	1 (0.51)	0 (0.00)	1 (0.40)
				ST2393	1 (0.51)	0 (0.00)	1 (0.40)
				ST2791	1 (0.51)	1 (1.75)	2 (0.79)
CC198	0 (0.00)	1 (1.75)	1 (0.40)	ST198	0 (0.00)	1 (1.75)	1 (0.40)
CC398	11 (5.64)	6 (10.53)	17 (6.75)	ST398	8 (4.10)	5 (8.77)	13 (5.16)
				ST804	2 (1.03)	0 (0.00)	2 (0.79)
				ST2103	0 (0.00)	1 (1.75)	1 (0.40)
				ST2392	1 (0.51)	0 (0.00)	1 (0.40)
CC2483	1 (0.51)	0 (0.00)	1 (0.40)	ST2483	1 (0.51)	0 (0.00)	1 (0.40)

MLST, multilocus sequence typing; CC, clonal complex; n, number of isolates.

## Discussion

The prevalence of MSSA nasal colonization among schoolchildren was 11.44% (195/1705). The rate was lower compared with that of secondary school students in Iraq (15.72%, 80/509) and university students in Italy (12.10%, 19/157) [25]. However, the rate was higher compared with that of university students in Nepal (7.33%, 22/300) [26], university students in China (10.08%, 212/2103) [27], and college students in Nigeria (8.65%, 16/185) [28]. Overall, the prevalence of MSSA nasal colonization in this study was moderate. With regard to MSSA environmental contamination in schools, the prevalence was 4.60% (57/1240), which was lower than that of MSSA environmental contamination in athletic facilities in the United States (32.00%, 40/125) [29], door handles of toilets and classrooms in secondary schools in Kenya (15.03%, 46/306) [30], and surfaces of computer keyboards in universities in the United States (62.50%, 15/24) [31]. Overall, the prevalence of MSSA contamination in the school environment of this study was not high. Nevertheless, there were positive prevalence relationships of MSSA isolates between the environment and schoolchildren. Of note, relevant departments should be aware of the hygiene in the entire school environment, including classrooms, toilets, and public places.

Our study contributes to the existing literature because we assessed a population with a high burden of obesity and MSSA colonization in which the association has not been examined in previous publications. Obesity was not a risk factor for MSSA nasal colonization among schoolchildren in the model of multivariate logistic regression analysis, which differed from other observed studies [10, 32]. Our study revealed that higher body fat percentage was related to increased frequency of *S*. *aureus* colonization [33]. Obesity may perturb the microbiome of the anterior nares, rendering them more conducive to colonization by *S*. *aureus* [10]. Accumulating evidence suggests that the human microbiome is influenced by and influences different pathological states [34, 35]. Other studies suggested that the interaction between site-specific microbiota and tissue-specific immune responses could hinder or propagate the proliferation of pathological species such as *S*. *aureus* [36, 37]. Family members who were healthcare workers was not an influencing factor for MSSA nasal colonization in this study. This finding needs to be further explored.

Given that the proportions of antibiotic resistance in MSSA isolates from schoolchildren and the environment were not different, the antibiotic resistance patterns of MSSA isolates from schoolchildren and the environment were similar, which might indicate the homology of MSSA isolates between schoolchildren and the environment.

The proportions of toxin genes in MSSA isolates were increased compared with other observed studies [27, 38], revealing increased toxicity of MSSA isolates in this study. Furthermore, the proportions of all toxin genes in MSSA isolates between schoolchildren and the environment were not significantly different, which might also indicate the homology of MSSA isolates between schoolchildren and the environment.

The dominant CC types and STs in this study were similar to other studies [27, 39]. The findings regarding 22 CC types and 71 STs of MSSA isolates were not different between schoolchildren and the environment. In addition, 17 STs were found in MSSA isolates from both schoolchildren and the environment. Dendrogram analysis also revealed the homologous relatedness of MSSA isolates between schoolchildren and the environment.

Therefore, according to the positive relationship of MSSA isolates, prevalence between schoolchildren and the environment and the homology of MSSA isolates between schoolchildren and environment based on phenotypic, genetic, and molecular characteristics, we suggest that both the decontamination of MSSA in the environment and the increasing hygiene of schoolchildren could be beneficial to reduce the prevalence of MSSA nasal colonization among schoolchildren.

One of the strengths of this study is that the sample size is relatively large and representative. Given considerable variability in BMI, it enabled us to make robust estimates of the relationships. The relatively large number of schoolchildren with a high response rate yields high statistical power to infer the characteristics of the study population. Moreover, this study is the first to elucidate the quantitative association of isolates between schoolchildren and the environment. We also performed phenotypic and molecular characterization of MSSA isolates to further assess the relationship.

This study also has several limitations. First, we cannot establish a cause and effect relationship because the study design is cross-sectional. Second, one of the strengths of the study is the use of multistage stratified cluster regression analysis, which overcomes the limitations of the standard regression analysis previously performed. Finally, given limited financial support, we could not offer decolonization measurements to schoolchildren, and whether such intervention would improve health of schoolchildren could not be scientifically proven.

## Conclusions

Despite these limitations, our findings suggest that both schoolchildren and the school environment were colonized with MSSA. Therefore, environmental decontamination, including enhancement of disinfection and cleaning, may be considered as strategies to decrease and prevent MSSA nasal colonization in schoolchildren.

## Supporting information

S1 Table STROBE checklist of this observational study. (DOCX)

S1 File An analysis plan of MSSA between schoolchildren and school environment. (DOCX)

S2 File A questionnaire of MSSA carriage in schoolchildren. (DOCX)

S1 Data A dataset of MSSA between schoolchildren and school environment. (XLS)
