# Supplementary material for: School environmental contamination of methicillin-sensitive Staphylococcus aureus as an independent risk factor for nasal colonization in schoolchildren: An observational, cross-sectional study
Source: PLoS One. 2018 Nov 30;13(11):e0208183. doi: 10.1371/journal.pone.0208183 (PMC6269093; doi:10.1371/journal.pone.0208183)
Supplement: S1 File — (DOCX) [file pone.0208183.s002.docx]

**S1 File. An analysis plan**

**Setting and study design**

This multistage stratified cluster cross-sectional study was conducted from March to August 2016 in eight elementary schools in Guangzhou, China. Schoolchildren were randomly selected using a multistage stratified cluster sampling design. Firstly, we randomly selected three administrative districts from the urban region and one administrative district from the rural region. Secondly, two elementary schools from each administrative district and eight schools were totally included. Thirdly, the number of class in grade four and five was proportionally and randomly selected. All schoolchildren that were with consent from each class were included in the study. Schoolchildren from grade four and five were selected due to their better compliance and less academic burden in China. Those who were absent or were excluded. The study was approved by the Ethics Committee of Guangdong Pharmaceutical University, and it was performed in accordance with the approved guidelines of the Declaration of Helsinki. All legal representatives of participants signed an informed written consent form.

We selected classrooms, toilets, and public places of each school for environmental sampling. We sampled five locations (floor, light switch, door handle, desk, chair, and air) of the classroom, four locations (floor, light switch, door handle, faucet, and toilet flush handle) of the two toilets (men’s and women’s), and three locations (handle of stairs, floor of stairs, and floor of corridor) of the public place from each class. Overall, we sampled 31 samples of each class. These locations are frequently touched by people, easily with skin contact, and amenable to cleaning and disinfecting. In order to avoid bias of sampling time, we performed sampling between two and four in the afternoon on weekdays.

**Questionnaires and sampling**

We collected information through questionnaires written by parents of schoolchildren. Demographic characteristics, personal characteristics, characteristics of family members, and household characteristics were collected. The questionnaire was first prepared in English and then translated into local language (Chinese), and back to English to ensure consistence. Sterile swabs moistened with sterile saline water were used to sample both nasal vestibules of the schoolchildren and surfaces of environmental locations by trained personnel. Floor, desks, and chairs were swiped approximately 10×10cm area because of their regular surfaces. Light switches, handles, and toilet faucets were swiped their entire area because of their irregular surfaces. Each swab was placed into a sterile tube with 7.5% sodium chloride broth, and the tubes were transported to the laboratory immediately at 4 °C during transportation.

We used the natural sedimentation method to collect air samplings. We put five mannitol salt agar plates on five desktops in each classroom (one plate in the center and four at the four corners of the classroom). After exposure of ten minutes, we closed the cover of plates and transferred them to the laboratory for further analyses. To ensure the acquisition of quality data and the quality of sampling, all investigators were trained by the principal investigator for three days before the survey and random checks were carried out by the principal investigator.

**Laboratory methods**

After 24 hours incubation at 36±1 °C, the swabs were then transferred to mannitol salt agar plates and incubated for 24-48 hours. Yellow colonies and/or colonies that had yellow zones on the mannitol salt agar plates were identified as *Staphylococcus aureus* (*S. aureus*) if there were visible as grape-like clusters and gram-positive bacteria under a microscope and were positive for the catalase, β-hemolysin, thermonuclease, and tube coagulase tests [1, 2]. We isolated one isolate per sample. *S. aureus* strain ATCC25923 was used for quality control.

All *S. aureus* isolates underwent cefoxitin susceptibility testing using the Kirby-Bauer disk diffusion method, following the Clinical and Laboratory Standards Institute guidelines 2015. *S. aureus* isolates that were sensitive to cefoxitin were identified as methicillin-sensitive *Staphylococcus aureus* (MSSA) isolates. All MSSA isolates were then underwent specific antibiotic susceptibility testing of 11 antimicrobial agents: cefoxitin (30μg), penicillin (10unit), linezolid (30μg), gentamicin (10μg), teicoplanin (30μg), erythromycin (15μg), trimethoprim-sulfamethoxazole (25μg), moxifloxacin (5μg), rifampin (5μg), chloramphenicol (30μg), tetracycline (30μg), and clindamycin (30μg). In erythromycin-resistant isolates, inducible clindamycin resistance was assessed using the D-zone test. MSSA strain ATCC29213 was used for quality control. The interpretation standard of the diameter of antibacterial circle can be found in Table 1.

Table 1 The interpretation standard of the diameter of antibacterial circle.

| Antimicrobial agents | Diameter (mm) | | |
| --- | --- | --- | --- |
|  | Sensitive | Intermediate | Resistant |
| Cefoxitin (FOX) | ≥22 | - | ≤21 |
| Penicillin (P) | ≥29 | - | ≤28 |
| Linezolid (LZD) | ≥21 | - | ≤20 |
| Gentamicin (CN) | ≥15 | 13-14 | ≤12 |
| Teicoplanin (TEC) | ≥14 | 11-13 | ≤10 |
| Erythromycin (E) | ≥23 | 14-22 | ≤13 |
| Trimethoprim-sulfamethoxazole (SXT) | ≥16 | 11-15 | ≤10 |
| Moxifloxacin (MXF) | ≥24 | 21-23 | ≤20 |
| Rifampin (RD) | ≥20 | 17-19 | ≤16 |
| Chloramphenicol (C) | ≥18 | 13-17 | ≤12 |
| Tetracycline (TE) | ≥19 | 15-18 | ≤14 |
| Clindamycin (DA) | ≥21 | 15-20 | ≤14 |

The DNA of all MSSA was extracted using Sodium dodecyl sulfate method [3] and were further tested to confirm the presence toxin genes [*Tst*, *Eta*, *Etb*, and Panton-valentine leukocidin (*Pvl*)] [4, 5] using polymerase chain reaction (PCR) assays. The PCR condition of *Pvl* was as follows: 3.0 μL of template DNA in a 25-μL final reaction volume containing 0.5 μL for the primers with the thermocycling conditions set at 94°C for 4 min, followed by 10 cycles of 94°C for 30 s, 60°C for 30 s, and 72°C for 45 s and 25 cycles of 94°C for 30 s, 52°C for 30 s, and 72°C for 45 s. The PCR condition of *Tst*, *Eta*, and *Etb* was as follows: 3.0 μL of template DNA in a 25-μL final reaction volume containing 1.0 μL for the primers with the thermocycling conditions set at 95°C for 5 min, followed by 30 cycles of 1 min of denaturation at 94°C, 1 min of annealing at 55°C, and 1.5 min of extension at 72°C; and a final extension step at 72°C for 10 min. More details of primer sequence of toxin genes can be found in Table 2.

Table 2 The primer sequence of toxin genes.

| Gene | Sequence (5’-3’) | Size of amplified product (bp) |
| --- | --- | --- |
| *Pvl* | ATCATTAGGTAAAATGTCTGGACATGATCCA | 433 |
|  | GCATCAAGTGTATTGGATAGCAAAAGC |  |
| *Tst* | TTCACTATTTGTAAAAGTGTCAGACCCACT | 180 |
|  | TACTAATGAATTTTTTTATCGTAAGCCCTT |  |
| *Eta* | ACTGTAGGAGCTAGTGCATTTGT | 190 |
|  | TGGATACTTTTGTCTATCTTTTTCATCAAC |  |
| *Etb* | CAGATAAAGAGCTTTATACACACATTAC | 612 |
|  | AGTGAACTTATCTTTCTATTGAAAAACACTC |  |

The Multilocus sequence typing (MLST) were carried using previously published primers and conditions [6]. Allelic profiles, clonal complex (CC) types, and sequence types (STs) were assigned using MLST database (http: //www. mlst.net). Singletons or members of a clonal complex were determined using the Based upon related sequence types algorithm (http://eburst.mlst.net). Dendrogram analysis was performed based on STs to determine the clonal relatedness and potential epidemiologic origin.

**Definitions of obesity**

Anthropometric measurements were taken using standardized techniques and calibrated equipment. Schoolchildren were weighted to the nearest 0.1 kg in light indoor clothing and bare feet or with stockings. The height was measured using a stadiometer; schoolchildren stood in erect posture without shoes, and the results were recorded to the nearest 0.1 cm. Measures were taken two times, and the average was considered in the analysis. Body mass index (BMI) was calculated as the ratio of weight in kilograms to the square of height in meters (kg/m^2^). The age- and gender-specific BMI cutoff points recommended by the Working Group on Obesity in China were used to define obesity [7].

**Statistical analysis**

Significance of the difference between proportions was using Pearson's Chi-square test for categorical variables. There were two clusters in the study. One was class cluster and the other was school cluster. We drew linear prediction plots to intuitively elucidate the dose-response relationship of MSSA isolates between nasal colonization among schoolchildren and environment in class or school clusters. The association between influencing factors and MSSA nasal colonization were examined using multivariable logistic regression models. We carried out the multivariable logistic regression analysis of all variables with a *P*-value of <0.05, and then removed variables that were not significant at this level. All statistical measures were estimated using survey data analysis methods from Stata package. A two-sided *P*-value for statistical significance was defined as *P*-value of < 0.05. All analyses were performed using STATA 14.2 (College Station, Texas, USA).

**Reference**

1. Evans JB. Anaerobic Fermentation of Mannitol by Staphylococci. J Bacteriol. 1947;54(2):266. PubMed PMID: 16561360.

2. Hill LR. The Adansonian classification of the staphylococci. J Gen Microbiol. 1959;20(2):277-83. doi: 10.1099/00221287-20-2-277. PubMed PMID: 13654722.

3. Liting Su JL, Xiaojian Guo, Fei Feng. Comparison of methods for DNA extraction in Staphylococcus aureus. Journal of Zhongkai University of Agriculture and Engineering. 2011;24(1):15-9. Epub 2011.3.

4. Jarraud S. Relationships between Staphylococcus aureus Genetic Background, Virulence Factors, agr Groups (Alleles), and Human Disease. Infection and immunity. 2002;70(2):631-41. doi: 10.1128/iai.70.2.631-641.2002.

5. McClure JA, Conly JM, Lau V, Elsayed S, Louie T, Hutchins W, et al. Novel multiplex PCR assay for detection of the staphylococcal virulence marker Panton-Valentine leukocidin genes and simultaneous discrimination of methicillin-susceptible from -resistant staphylococci. J Clin Microbiol. 2006;44(3):1141-4. doi: 10.1128/JCM.44.3.1141-1144.2006. PubMed PMID: 16517915; PubMed Central PMCID: PMCPMC1393128.

6. Enright MC, Day NP, Davies CE, Peacock SJ, Spratt BG. Multilocus sequence typing for characterization of methicillin-resistant and methicillin-susceptible clones of Staphylococcus aureus. Journal of clinical microbiology. 2000;38(3):1008-15. PubMed PMID: 10698988; PubMed Central PMCID: PMC86325.

7. Group of China Obesity Task F. [Body mass index reference norm for screening overweight and obesity in Chinese children and adolescents]. Zhonghua Liu Xing Bing Xue Za Zhi. 2004;25(2):97-102. PubMed PMID: 15132858.
