# Supplementary material for: School environmental contamination of methicillin-sensitive Staphylococcus aureus as an independent risk factor for nasal colonization in schoolchildren: An observational, cross-sectional study
Source: PLoS One. 2018 Nov 30;13(11):e0208183. doi: 10.1371/journal.pone.0208183 (PMC6269093; doi:10.1371/journal.pone.0208183)
Supplement: S2 File — (DOCX) [file pone.0208183.s003.docx]

**Consent Form**

We are currently conducting an investigation on the bacterial colonization and relevant factors in schoolchildren. The investigation is of non-commercial purpose. We guarantee that all the information will be confidential and safe and will not disclose to anyone without the consent of the respondent.

If you agree your child to participate in this investigation, please sign your name:

**Investigation of bacterial colonization and relevant factors**

**among schoolchildren in Guangzhou**

School: Grade: Class: Name:

Gender:(0) Male (1) Female Birthday: Age:

Living region: (0) Urban (1)Rural

Height: cm Weight: kg

**Personal characteristics (within a year)**

1.Have he/she ever had antibiotic use? (0) No (1)Yes

2. Have he/she ever had skin infections? (0) No (1)Yes

3. Have he/she ever had allergic diseases? (0) No (1)Yes

1. Have he/she ever had upper respiratory tract infections? (0) No (1)Yes
2. Have he/she ever had surgical interventions? (0) No (1)Yes
3. Have he/she ever had outpatient services? (0) No (1)Yes
4. Have he/she ever had hospitalizations? (0) No (1)Yes
5. Have he/she ever shared towel with others? (0) No (1)Yes
6. Have he/she ever had physical exercise every week? (0) No (1)Yes
7. Have he/she ever washed hands more than six times everyday? (0) No (1)Yes
8. Have he/she always washed hands after using toilets? (0) No (1)Yes
9. Have he/she gone to school by public transportations? (0) No (1)Yes

**Characteristics of family members (within a year)**

1. Have he/she ever had antibiotic use? (0) No (1)Yes
2. Have he/she ever had skin infections? (0) No (1)Yes
3. Have he/she ever had upper respiratory tract infections? (0) No (1)Yes
4. Have he/she ever had allergic diseases? (0) No (1)Yes
5. Have he/she ever had hospitalizations? (0) No (1)Yes
6. Is there any healthcare workers? (0) No (1)Yes
7. Is there any current smokers? (0) No (1)Yes

**Household characteristics**

1. Is there more than three people in your family? (0) No (1)Yes
2. Single child: (0) No (1)Yes
3. Currently live with pets: (0) No (1)Yes

The end. Thanks for your cooperation.

**知情同意书**

目前我们正在联合进行一项关于我市小学生细菌感染及相关因素情况的调查。该调查非商业用途，我们保证被调查者资料的保密性和安全性，非经被调查者同意，不会透漏给任何人。

若您同意您的孩子（被监护人)参加此次调查，请签上您（监护人)的名字：

**广州市小学生细菌定植及相关因素情况调查表**

您好！本次调查的目的是了解我市小学学生细菌定植及相关因素情况，您所填写的任何您孩子的信息都不会公开，请您如实填写，选择题请在选项打钩“√”或者把选项写在题号前面。

学校： 年级： 班别： 姓名：

性别：（0)男 （1)女 出生日期： 年 月 日 年龄: （岁)

居住地区：（0)城区 （1)郊区

身高： 厘米 体重： 公斤

**您的孩子情况**

1.您的孩子近一年内有无服用抗生素：（0)无（1)有

2.您的孩子近一年内有无皮肤感染史：（0)无（1)有

3.您的孩子近一年内有无过敏性疾病史：（0)无（1)有

1. 您的孩子近一年内有无上呼吸道感染史：（0)无（1)有
2. 您的孩子近一年内有无外科手术史：（0)无（1)有
3. 您的孩子近一年内有无门诊或医院就医史：（0)无（1)有
4. 您的孩子近一年内有无住院史：（0)无（1)有
5. 您的孩子近一年内有无与其他人共用毛巾（浴巾)：（0)无（1)有
6. 您的孩子是否每周进行体育锻炼：（0)否（1)是
7. 您的孩子是否每天洗手次数>6：（0)否（1)是
8. 您的孩子是否每次便后都洗手：（0)否（1)是
9. 您的孩子是否使用公共交通工具上学：（0)否（1)是

**家庭成员情况**

1. 家庭成员近一年内有无服用抗生素：（0)无（1)有
2. 家庭成员近一年内有无皮肤感染史：（0)无（1)有
3. 家庭成员近一年内有无上呼吸道感染史：（0)无（1)有
4. 家庭成员近一年内有无过敏性疾病史：（0)无（1)有
5. 家庭成员近一年内有无住院史：（0)无（1)有
6. 家庭成员中有无人在医疗卫生系统工作：（0)无（1)有
7. 家庭成员中有无人吸烟：（0)无（1)有

**家庭情况**

1. 家庭人数是否大于3：（0)否（1)是
2. 是否独生子女：（0)否（1)是
3. 家中有无养宠物：（0)无（1)有

调查到此结束，谢谢您的合作！
